# Through the lens of Good Participatory Practice: Findings and lessons learned from the healthcare worker subcommittee of the COVID-19 Healthcare Worker Exposure Response and Outcomes Registry

**DOI:** 10.1017/cts.2024.668

**Published:** 2024-12-12

**Authors:** Mei Lin Chen-Lim, Jayne F. Koellhoffer, Kisha Batey Turner, Martha Summerlin, Anoop George, Eileen Handberg, Daryl Lawrence, Syed Hasan Naqvi, Emily O’Brien, Lauren Cohen, Patty McAdams, Laura Webb, Renee Leverty

**Affiliations:** 1 College of Nursing, Thomas Jefferson University, Philadelphia, PA, USA; 2 Doylestown Hospital, Doylestown, PA, USA; 3 Vanderbilt University Medical Center, Nashville, TN, USA; 4 Duke Clinical Research Institute, Durham, NC, USA; 5 Temple University Health System, Philadelphia, PA, USA; 6 University of Florida, Gainesville, FL, USA; 7 University of Missouri, Columbia, MO, USA

**Keywords:** Bidirectional engagement, clinical research, participant advisory committee, diversity, equity, inclusion, Good Participatory Practice

## Abstract

Participant representation, including the Good Participatory Practice guidelines, in the design and execution of clinical research can profoundly affect research structure and process. Early in the COVID-19 pandemic, an online registry called the Healthcare Worker Exposure Response and Outcomes (HERO) Registry, was launched to capture the experiences of healthcare workers (HCWs) on the pandemic frontlines. It evolved into a program that distributed COVID-19-related information and connected participants with COVID-19-related research opportunities. Furthermore, a subcommittee of HCWs was created to inform the COVID-19-related clinical research, engagement, and communication efforts. This paper, coauthored by the HERO HCW subcommittee, describes how it was formed, the impact of community participation on the HERO Registry and Research Program, reflections on lessons learned, and implications for future research. Engagement of the HCW Subcommittee resulted in representing their lived experience and ensured that their perspectives as HCWs were incorporated into the HERO Research. The strategies not only supported recruitment and retention efforts but also influenced the HERO research team in framing research questions and data collection pertinent to the participant community. This experience demonstrated the importance of having participants’ input as expert advisors to an investigative team in their research efforts during a global health emergency.

## Introduction

Participant or community engagement in research, including participatory action research (PAR), are not new concepts in clinical trials but remain rare in practice [1–4]. The struggles of healthcare workers (HCWs) early in the pandemic have been well published [5–7], yet HCWs were also involved in participatory research aimed at tackling COVID-19. Finding treatments and a protective vaccine for COVID-19 was at the forefront of public health efforts, creating an urgent need to quickly coordinate clinical research efforts on local, national, and international levels early in the pandemic. This urgency extended to frontline HCWs who struggled with the diagnosis and management of COVID-19-infected patients. HCWs also had to grapple with the critical lack of personal protective equipment (PPE) and fear for their own safety and that of loved ones. Although the needs and concerns of HCWs evolved over the course of the pandemic, there was often little opportunity for HCW groups to voice lived experiences at the moment.

Thus, while efforts were underway to find treatments and vaccines for public health, HCWs were also enlisted as research participants to learn about their experiences and struggles during an active crisis. A national registry, the Healthcare Worker Exposure Response and Outcomes (HERO) Registry, opened to HCWs across many roles, focused on the experiences of HCWs, shed light on their experiences during the pandemic, and enlisted participation in COVID-19-related clinical research. The HERO Principal Investigators, who launched the HERO Registry, also formed a subcommittee to increase participant input through Good Participatory Practice (GPP) guidelines [4,8]. This subcommittee of HCW Registry members, representing various HCW roles across the U.S., informed the continuing structure, research, and design of the HERO Registry Program. As trial participants and community stakeholders of the HERO Registry, HCW subcommittee members voiced the needs and experiences of HCWs nationally based on individual diverse backgrounds during the pandemic. The HCW subcommittee and HERO Registry team embraced the principles of mutual respect, trust, transparency, and accountability while maintaining community stakeholder autonomy over the course of the subcommittee work. The purpose of this paper is to describe how the HCW subcommittee was formed, how community participation impacted the HERO Registry and Research Program, lessons learned, and implications for future research.

## How the HCW subcommittee was formed

### HERO Registry

In March 2020, the Duke Clinical Research Institute (DCRI) received funding from the Patient-Centered Outcomes Research Network (PCORnet), a subsidiary of the Patient-Centered Outcomes Research Institute (PCORI), to create a novel online registry of HCWs. The goal of the HERO Registry was to bring together HCWs from across the country and centralize outreach and data collection regarding the experiences of HCWs during the pandemic. HERO defined HCWs as anyone who worked in a setting where people received healthcare, including nurses, therapists, physicians, laboratory workers, food service workers, environmental service workers, interpreters, emergency responders, transporters, and others.

**Figure 1.**
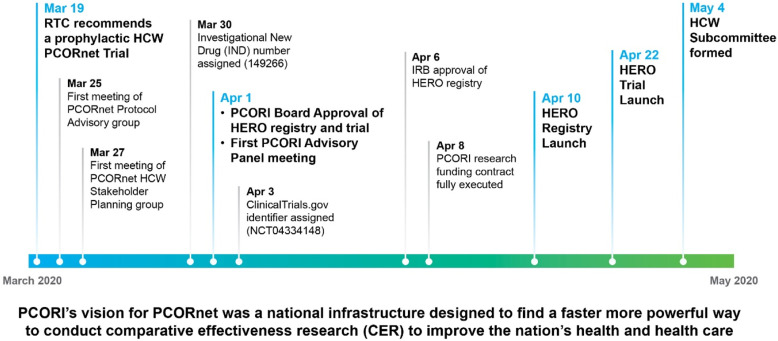
Timeline of the first weeks of the creation of the HERO Registry, HERO-HCQ trial, and HCW subcommittee. HCW = healthcare worker; HERO = Healthcare Worker Exposure Response and Outcomes; IRB = institutional review board; PCORI = Patient-Centered Outcomes Research Institute; PCORnet = Patient-Centered Outcomes Research Network; RTC = Research Transformation Committee, PCORI.

By collecting data from frontline HCWs in real time, the HERO Registry created an opportunity to rapidly generate data on issues relating to the protection of HCWs’ health and well-being, including topics such as stress, burnout, moral injury, anger, COVID-19 testing, PPE usage, and racial and ethnic disparities [9,10]. In addition, HERO Registry participants were invited to join COVID-related clinical research. This research included HERO-HCQ, a randomized controlled clinical trial testing hydroxychloroquine (HCQ) as a pre-exposure prophylaxis against COVID-19 infection [11], and HERO-TOGETHER, a 2-year observational study of registry participants’ experiences after receiving a COVID-19 vaccine [12]. The HERO Registry was launched on April 10, 2020. The HERO-HCQ trial began recruitment shortly thereafter, on April 22, 2020 (Figure 1). Findings from the HERO Registry are available from Forrest et al. [9] and Friedland et al. [10]. PCORnet provided access to 8 clinical research networks across the U.S. as hubs for sharing awareness and recruiting to both the HERO Registry and the HERO-HCQ trial.

### Participant-engaged research

The HERO Registry launched within a month of the national lockdown in the U.S., and the HCW subcommittee was formed in May 2020. See Forrest et al. [9] for details on the launch of the registry. Patient- or community-engaged research is a requirement for PCORI funding, and there are many ways to adopt these principles or strategies [2,13], such as community-based participatory research (CBPR) [14,15], PAR [3], or GPP [4,8]. CBPR and PAR, which would have required various HCW engagements from the initial planning and design, were not feasible at the time due to the urgency to launch the program and capture the experiences of the frontline HCWs. Thus, in line with GPP, creating an HCW subcommittee was necessary as there were no existing relationships among the HERO research team to provide HCW knowledge and experience across various roles that could inform the research regarding inclusive outreach, accessible design, and understanding value for participants.

### Good Participatory Practice

The idea of GPP was formally proposed in 2007 by the Joint United Nations Programme on HIV/AIDS (UNAIDS) to address ethical and equality issues arising from HIV prevention trials [4]. GPP guidelines were published to instruct those involved in the design and implementation of biomedical human immunodeficiency virus (HIV) prevention trials on how to effectively engage community stakeholders and collaborate with them during all trial phases. Collaboration between researchers and the individuals or groups with a stake in the trial outcome was found to increase innovation, improve study participation and equity, and ultimately enhance and broaden the applicability of study results. GPP is now an integral part of HIV prevention research [16,17]. Community partner engagement and GPP have been implemented in many other research contexts as well, ranging from Ebola vaccine trials [18] to tuberculosis drug trials [19] to an after-school social intervention aimed at reducing school dropout among adolescent girls in South Africa [20]. Partner engagement reflecting community or individual lived experiences is also viewed as crucial for addressing new, emerging pathogens for which limited medical interventions are available and for which misinformation and/or social taboos may be rampant. In 2016, the World Health Organization published GPP guidelines for trials involving such pathogens [8] and later adapted a toolbox for COVID-19 [21]. These principles have been particularly important when applied to research on COVID-19 and have provided a path to foster trust between research teams and study participants during the global pandemic [22–25].

### Initiation of the HCW subcommittee

At the center of the engagement initiatives for the HERO Registry, the HERO HCW subcommittee was created to advise the HERO research team by providing insights and perspectives based on their lived experiences as representatives of the healthcare workforce during the COVID-19 pandemic. HCWs make up 14% of all U.S. workers; they differ in terms of their roles, cultural identities, workplace settings, and communities of residence [26]. The HERO Registry was open to all U.S. HCWs. Therefore, to reflect the diversity among the HCWs in the registry, an effort was made to choose HCW subcommittee members of diverse racial backgrounds, ages, genders, HCW roles, geographic locations, facility types, and experiences.

**Figure 2.**
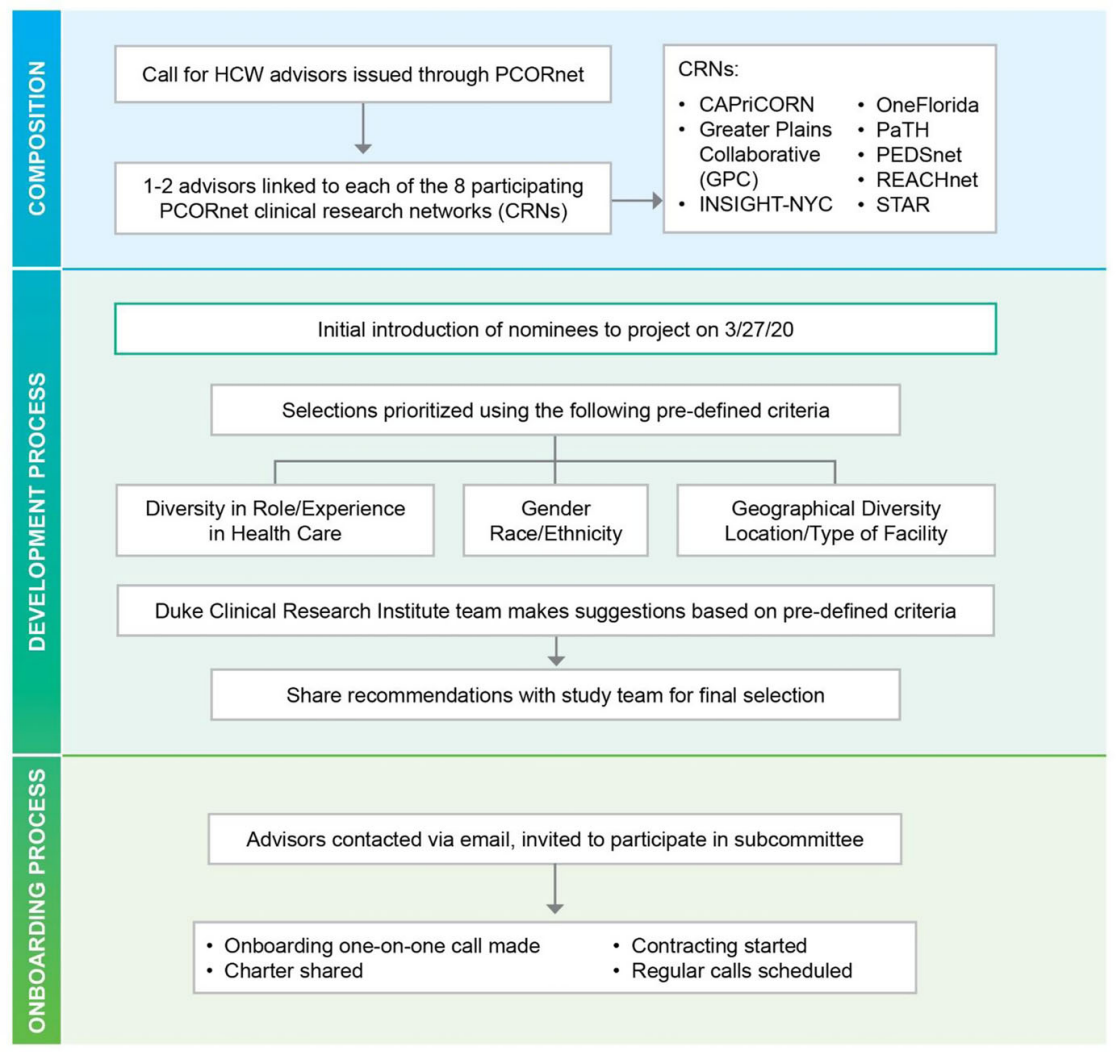
Process of nomination and selection for the members of the HCW subcommittee. CAPriCORN = Chicago Area Patient-Centered Outcomes Research Network; CRN = clinical research network; HCW = healthcare worker; HERO = Healthcare Worker Exposure Response and Outcomes; PaTH = Path Towards a Learning Health System; REACHnet = Research Action for Health Network, Science, Technology and Research partnership.

HERO research leadership mainly comprised of researchers and academics, identified a need for representation from HCWs. A call for advisors to serve on the HCW subcommittee was issued through PCORnet to the eight clinical research networks in March 2020 with the goal of forming the subcommittee and starting work with the HERO Registry as quickly as possible given the rapidly emerging pandemic. Potential candidates either applied directly for membership or were nominated by another contact in their network. Tasked by the HERO executive committee to form the HCW subcommittee, a team composed of engagement specialists trained in bidirectional engagement, inclusive partnerships, and co-learning principles reviewed applications and conducted phone interviews with individual applicants as part of the selection process. The phone interviews also enabled applicants to understand the role and expectations of subcommittee members. One applicant from each of the eight PCORnet clinical research networks was recommended by the engagement team to serve on the subcommittee. The HERO leadership reviewed these recommendations and contacted the selected members via email to formally invite them to participate in the HCW subcommittee. The process for nomination and selection is outlined in Figure 2.

The HCW subcommittee was composed of non-licensed (*n* = 3) and licensed (*n* = 5) HCWs: an environmental service worker, a paramedic, a medical technician, a respiratory therapist, nurses, a clinician, and a hospital administrator in pediatric and adult care health settings. The racial and ethnicity distribution included three members who identify as non-Hispanic Asian, three members identifying as non-Hispanic Black/African American, and two members identifying as non-Hispanic White. Members were from the Northeast, Southeast, and Midwest regions of the US. Two subcommittee members, a respiratory therapist and a medical laboratory scientist, were elected as co-chairs. The co-chairs also became members of the HERO executive committees, to create conduits for bidirectional communication with HERO leadership. Their liaison role aimed to ensure the adoption of priorities defined by the HCW subcommittee.

A charter was created to outline the activities, purpose, and responsibilities of HCW subcommittee membership. Contracts between the members and the DCRI were formalized, and each HCW subcommittee member received an honorarium for their time. Committee membership was renewed on an annual basis.

### HCW responsibilities

The HCW subcommittee was primarily responsible for providing recommendations to the HERO leadership on how to best engage with and understand the priorities of the members of the HERO Registry. Specific input provided by the HCW subcommittee included the following:

• Helping prioritize research topics

• Identifying privacy concerns

• Recommending approaches to ensure the enrollment and retention of HERO Registry members with the registry itself and with COVID-related clinical trials

• Advising on ensuring diverse outreach and enrollment into the registry

• Creating connections and insights to organizations and association partnerships

• Messaging and delivering recruitment & retention material and study communications

• Proposing methods of disseminating research findings to multi-stakeholder audiences

The HCW subcommittee met virtually on a monthly basis starting in May 2020 and lasting through August 2022. The typical meeting included check-ins from subcommittee members about their experiences on the front line, HERO Program updates, and bidirectional dialog regarding the priorities of the HCW subcommittee and the current initiatives of the HERO Registry Program and research team. The HCW subcommittee members provided up-to-date insights on the impact of COVID-19 as an evolving healthcare crisis given that all of the members were HCWs. Study leadership attended meetings with the HCW subcommittee to discuss the latest developments in the COVID-19 scientific landscape and pandemic trends affecting HCWs.

The GPP principles of mutual respect, trust, transparency, and accountability were intentional from the beginning of the HCW subcommittee and the HERO Registry and research team. As an example of mutual respect and transparency, subcommittee members were also placed in governance roles as key members of all HERO committees, including the HERO executive, registry, publication, and ancillary studies committees. Two of the HCW subcommittee members were a part of HERO executive committee in an effort to integrate subcommittee into overall project design. (Supplementary material, HERO Org Chart Figure.) Over the course of the HCW subcommittee engagement, HCW voices and experiences were embedded within the operational structure to guide and influence the HERO Registry’s design, development, and future direction. From the initiation, the HERO research team recognized the importance of maintaining HCW community stakeholder autonomy. The subcommittee did not speak for HCWs as a whole but was able to voice individual concerns and experiences at every meeting, sharing thoughts on research proposals, approaches, or outreach with the intent to broaden applicability, equity, and inclusion across roles or demographics. Initiatives of the HCW subcommittee included:

• Engaging HCW and HCW groups with authenticity to inform and enhance inclusive study recruitment and retention strategies

• Establishing a shared governance model, in which HCWs partnered with clinician-scientists in the research process

• Establishing diverse membership in committee and group work to ensure voices were heard from multiple perspectives

• Developing multifaceted approaches that combined engagement tools including systems for returning value to HERO Registry participants

• Implementing study strategies and metrics for enhanced HCW engagement and adoption of HCW advisor-directed priorities

### HCW subcommittee experience

At the end of year 1, a survey was conducted with all HCW Subcommittee members to understand members’ experiences and assess the effectiveness of the engagement. Additionally, near the end of the HCW subcommittee engagement, six semi-structured interviews were conducted via phone and Zoom with available subcommittee members from February 4, 2022, through March 1, 2022. The HCW subcommittee member interviews were conducted and analyzed by a non-partisan team from the coordinating center. All members disclosed they were HERO Registry participants during the HCW subcommittee interviews. Table 1 highlights a reflective summary of the HCW subcommittee member experience.

**Table 1.** Healthcare worker (HCW) subcommittee interview findings

Interview Questions	Summary
How long have you been involved with the HERO Registry? Why did you decide to become involved with the HERO Registry? How do you describe your role as a member of the HCW Subcommittee? Do you feel as though your interest in joining the HERO Registry is being satisfied? (Question suggested by subcommittee member) How can the subcommittee be utilized better? (Question suggested by subcommittee member) What changes could be made to the subcommittee structure? (Question suggested by subcommittee member In your own words, how do you describe the HERO Registry? How do you describe the HERO Registry’s purpose? What, if any, strategies have you tried to recruit new Registry participants? What, if any, strategies have you tried to recruit diverse Registry participants, with respect to their racial/ethnic identity and/or their professional role? Thinking about the HERO Registry’s digital presence and messaging, how could it be improved to attract more diverse participants In the coming years, what do you believe will be the value of the HERO Registry?	All members of the HCW subcommittee have been involved with the HERO Registry since mid-2020, which corresponds with either their direct involvement or interest in the HERO Hydroxychloroquine (HERO-HCQ) trial. Initial excitement about joining the HERO Registry has waned, though subcommittee members remain enthusiastic about its value as a platform. Subcommittee members feel responsible for providing input to the HERO steering committee; they feel it is important to voice concerns shared by others in their profession or with their same racial/ethnic identity. While the platform establishes a connection to other HCWs, subcommittee members feel it can do more to generate broad community support and amplify HCW voices on issues beyond COVID-19. Subcommittee members enjoy participating on the subcommittee, and they want the steering committee to utilize their expertise. The subcommittee believes its members should reflect the diversity of HCWs that the HERO Registry hopes to attract. Subcommittee members suggest that the subcommittee’s purpose be reassessed. In addition to refocusing the subcommittee, it may help determine the value of adding new members. When asked about the purpose of the HERO Registry, subcommittee members describe *who it is for* and *the opportunity for research exposure*. Few of the subcommittee members describe a “strategy” for recruiting new, diverse HERO Registry participants. Subcommittee members only express certainty that their close contacts have joined the HERO Registry; they are unsure of the effectiveness of their broader recruitment efforts. Several subcommittee members describe the HERO website as catering more to academics. Subcommittee members recommend clear, tailored messaging about the value of participation beyond research. While there is uncertainty about the HERO Registry’s future, subcommittee members believe there is potential to further connect participants and address HCW concerns.

*Notes:* Subcommittee members did not necessarily respond to each question, nor were questions asked in the same order. While points above may be attributed to certain subcommittee members, the list captures their collective sentiment.

## The impact of the HCW subcommittee

The HCW subcommittee impacted a wide range of initiatives throughout the HERO Registry Program from recruitment to generation of the HERO TOGETHER research. Documentation from all HCW Subcommittee meetings, activities, and impact were captured in the HERO Engagement Plan which was a living document throughout the project. Table 2 highlights the areas of impact and the outcomes. These efforts led to connections beyond the usual reach of the HERO research team and had an influence on future research programs.

**Table 2.** Examples of healthcare worker (HCW) subcommittee engagement with the HERO Registry Program and Research

HERO Program and Research Areas of Need	HCW Subcommittee Engagement	Outcomes
Research communication & educational outreach	For the HERO-HCQ Trial, the HCW subcommittee provided strategic educational outreach (e.g., outlets and messaging internal and external to the health system relevant to HCWs during the peak of the pandemic) to clarify and offset contradictory and confusing messaging in the popular press about HCQ.	Clarified published messaging regarding HCQ data. Facebook Live Town Hall was held to discuss HCQ and the trial. The HCW subcommittee chair acted as co-moderator for this town hall.
Research recruitment: Local efforts	During low or plateaued enrollment of the HERO research, members became site champions by engaging in outreach within their organizations through departmental meetings, information/materials sharing, communication about the research and goals, and sharing of experiences with research participation.	Increased awareness of HERO participation, which provided the ability to answer questions directly from potential participants and convey questions or concerns related to the research to program leadership from other HCWs.
Recruitment: Additional outreach	In addition to the local efforts within the subcommittee member’s organization, members of the subcommittee identified and introduced professional organizations or societies within disciplines or communities for targeted outreach.	Examples include the addition of specific campaigns to Emergency Medical Services (EMS) and Respiratory Therapy organizations and the American Academy of Family Physicians.
Recruitment and retention strategies	Recruitment materials: Subcommittee members reviewed materials and provided feedback to the HERO engagement team through an individual emails or surveys and monthly group discussion meetings regarding messaging on the website and in the program promotional toolkit. Diversity, Equity and Inclusivity (DEI) considerations: The subcommittee members suggested approaches to enhance DEI efforts to ensure robust HCW engagement. During advertisement efforts related to the program and research, members advised on messaging and images with an inclusivity mindset using images of HCWs of various races, ethnicities, and roles. Advertisements were revised to remove inadvertent stereotypes (e.g., drawings of faces with slanted eyes). The members advocated for valuation of participant time through compensation and results sharing.	Revision of HERO Registry and HERO-HCQ materials to include a portrayal of diverse HCW roles, race, ethnicity, and gender on the website and in the promotional toolkit. An outreach campaign featuring an HCW subcommittee member who worked in environmental services. The campaign included a blog, social media posts, and a flyer containing a quote and image, calling attention to the unsung heroes and key members of the healthcare team. Direct letter campaign to all U.S. skilled nursing facilities with over 50 beds, informing personnel of the HERO Registry opportunity. Participant compensation included in the HERO Registry and HERO-HCQ.
Dissemination of research results	The members provided critical feedback regarding the return of serology results and lay summaries for HERO-HCQ, emphasizing health literacy principles. The members recommended general principles for the return of results across the HERO Program, including abbreviated messaging with a focus on graphics to display information in a clear and concise way and to simplify distribution to colleagues and via social media.	Implementation of the following changes to HERO-HCQ regarding the return of serology results: • Notification email sent to participants in advance of results • Supporting documentation for results simplified to an 8^th^ grade reading level and modified to be written in active voice. • Potential interpretations tailored to the participant’s actual test results • Text added to express thanks and/or well wishes; study contact information included in case of questions. • Revision of the HERO-HCQ lay summary for health literacy and accessibility. • Creation of videos to share the findings of “Hot Topic” surveys.
Additional research questions to address HCWs’ concerns during the pandemic	The HERO program sought to meet the needs of the HCWs through its research during the pandemic. The HCW subcommittee, as a community stakeholder group, identified timely research questions of interest for the HERO Registry to reflect the evolving concerns of HCWs across the country. Members provided input to guide decision-making about research question prioritization, wording, and response options. Topics of concerns discussed during the pandemic included the following: • Variation in vaccination rates in the work setting and the impact on workload and responsibilities • Financial implications for employers, including furloughs or hiring freezes • Nurses leaving the profession • Understanding long-term immunity • Variations in personal protective equipment use, access, and availability among differing work environments, geographic regions, and HCW roles • Moral injury caused by an inability to support patients’ well-being due to limited visitation or an inability to have family at the bedside at the end of life HCW burnout due to exhaustion, constant evolution of rules and regulations, moral injury, challenges in work/life balance, and risk of spreading infection to family The discussion of these concerns led the HERO research team to conduct several “Hot Topic” surveys to engage HERO Registry participants based on recommendations (*Is Anger Affecting Healthcare Workers?* and *Who is Considering Leaving Health Care*?).	Sharing of the results of “Hot Topic” surveys with participants by the HERO research team via newsletters and blogs to demonstrate HCWs’ experiences across the US. Pipeline and active funded research titles generated from the HCW subcommittee and “Hot Topic” surveys: “Facility Characteristics and Healthcare Worker Outcomes during the COVID-19 pandemic: Results from the HERO registry” “Comparing Moral Injury Rates and Covariates in Two Samples: Post-9/11 Combat Veterans and COVID-19 Frontline Healthcare Workers” “Healthcare worker burnout during the COVID-19 pandemic: Insights from the HERO registry” “Gender and intention to leave healthcare during the COVID-19 pandemic among U.S. healthcare workers: A cross-sectional analysis of the HERO Registry” “Vaccinations and boosters over time”
Sharing Learning Broadly	Subcommittee co-created and coauthored abstract/poster titled “HERO Registry: A Multipronged Engagement Approach.”	Poster presented at PCORI 2020 Annual Meeting

*Notes:* The HCW subcommittee represented the community stakeholder group of the HERO Registry Program and trial participants of the research according to the Good Participatory Practice Guidelines [21]. The table provides descriptions of how the HCW subcommittee met the needs of the HERO Registry and research program. The results column includes outcomes or impacts as a result of the HCW subcommittee engagement that improved the efforts of the HERO Registry Program and Research.

EMS = emergency medical services; HCQ = hydroxychloroquine; HCW = healthcare worker; HERO = Healthcare Worker Exposure Response and Outcomes.

### Influence on recruitment, retention, and educational outreach

The HCW subcommittee’s insights informed messaging and the delivery of recruitment and retention communications for the HERO Registry and the two associated clinical trials. Subcommittee feedback prompted changes in recruitment materials and social media kits to increase clarity in the registry and trials and to increase diverse representation of HCWs in promotional materials. Additionally, the public-facing website was reconstructed to improve user experience and increase the visual representation of people of color and diverse HCW roles. Several subcommittee members created recruitment videos to encourage membership in the HERO Registry. Finally, messaging around the mRNA COVID-19 vaccines was created to support vaccination efforts with input from the subcommittee members. See Table 2 for additional details.

### Engaging national HCW organizations, unions, and associations

A communication plan was developed to directly engage HCW stakeholder organizations in the HERO Registry. The engagement team, research leadership, and HCW subcommittee members leveraged their contacts and previously formed trusted relationships and reached out to 47 healthcare organizations to engage members in the HERO Registry.

An example of such outreach included the SEIU Local 2015 Los Angeles, California Facebook Live Event with Dr Naggie, a HERO Investigator and infectious disease specialist, as a guest panelist. Dr Naggie discussed the HERO Registry as well as the HERO-recruited vaccine trial, HERO Together; the video has since been viewed over 1,600 times. An example organization that was engaged by HERO outreach efforts is The American College of Physicians, Inc., which promoted the HERO Registry to its two boards and included information on HERO in its weekly news email.

### Influence on research initiatives

Feedback from the subcommittee members also identified priority topics for HCWs at different times during the pandemic. In addition, the members stressed the importance of focusing on research outcomes most meaningful to HCWs at the time and ongoing approaches to ensure robust engagement and value for participating in HERO. Specific forms of engagement by the HCW subcommittee that translated to direct changes in the HERO Registry or the HERO-HCQ/HERO-Together trials’ inclusive outreach, accessible design, or efforts to create mutual value are described in Table 2.

### Exemplar of subcommittee member contribution

In February 2021, an outreach campaign featuring a subcommittee member who worked in environmental services was launched in response to feedback from the HCW subcommittee that many individuals who did not perform direct patient care did not identify themselves as “healthcare workers.” The HCW subcommittee member co-developed all material content, including a photo and the following quote: “We just want to keep people safe, and while doing our job to clean and disinfect surfaces to reduce the spread of the virus, we face the risk of COVID-19 ourselves and put our families at risk. By joining the HERO Registry, we can share our experiences on the front lines and participate in research that can make a difference in this crisis.” This campaign utilized a blog, social media posts, and a flyer to highlight key members of the healthcare team whose roles are often overlooked, and these materials were shared broadly through social media and PCORnet Clinical Research Networks.

## Reflections on lessons learned

With approximately 22 million HCWs in the U.S., creating a truly representative group to give voice to the common experience of HCWs during the COVID-19 pandemic was difficult. The HERO subcommittee was formed rapidly at the beginning of the COVID-19 pandemic, with nominations made only through the eight clinical research networks. This meant that the western U.S. was not represented geographically within the subcommittee. In addition, the members had defined roles in healthcare and may not have had an accurate picture of the experiences of other HCWs nationally. The work would have been better informed by a larger group of HCWs that included more geographic diversity, more types of HCW roles, and broader diversity in ethnic/cultural identity. In retrospect, the subcommittee should have been expanded to include such representative members over time if such members were not initially available. Such changes or additions should be made if another national community of HCWs is established for future research endeavors.

Additionally, although a survey of subcommittee members halfway through their two-year membership showed that members had positive feedback about their participation, members also commented on their desire to accomplish more with the registry. Six out of eight members completed the survey responses. Results showed that members felt that they had enough information about the topic areas to participate effectively in the subcommittee; that the engagement team had a clear understanding of members’ expertise, strengths, and roles; that open communication was fostered; that information was presented in understandable ways; and that co-learning occurred. Yet, both the year 1 survey and year 2 interviews of the HCW subcommittee members shed light on several concerns. First, they indicated that there were no specific tasks for members to complete, aside from sharing insights during or in between meetings. In addition, outside of the two clinical trials, there was a lack of clear goals for questions the research was aimed to address. Finally, the members felt that they had more of a consultative rather than collaborative role with the HERO executive committee and the HERO Registry as a whole. Collaboration may have been better fostered with a clearer picture of the tasks and goals of the HERO Registry project where subcommittee members could lend their expertise and perspectives to specific research objectives. Future research, including a community partner group such as the HCW subcommittee, should include periodic reassessment of membership experience to strengthen GPP principles and the research representation of the participants and its community.

## Implications for future research

An engagement strategy that centers equity through inclusive committee structure and pathways of influence can generate a balanced perspective and directly influence various aspects of a program [27,28]. Lessons learned from the HCW subcommittee’s work on HERO include the following:

- Adopt GPP guidelines [21] with the population being researched partnering with clinician-scientists to develop and implement the research process, especially in the setting of an emergent disease or during a global health emergency.
- Establish diverse membership in committees to ensure that voices are heard from multiple perspectives [29,30].
- Employ agile strategies to include representative voices; initial engagement plans must include frequent assessments and adaptability to ensure that the engagement strategy is effective and that the communities experiencing the greatest impact are partners in the research. Examples include seeking partnerships with organizations that serve racially/ethnically diverse groups, such as the National Association of Hispanic Nurses; or creating ways to collect diverse perspectives, such as shorter-term focus groups or listening sessions.
- Co-create opportunities for growth, mutual value, and enhanced involvement based on partners’ interests or expertise. Examples include being members of executive committees, coauthoring manuscripts or posters, leading virtual or in-person events, or being ambassadors for programs at a local or national level.
- Implement strategies to enhance HCW engagement and track the adoption of HCW partner-directed priorities.

## Conclusion

GPP has enhanced clinical research in many areas and is especially important in trials dealing with new/emergent diseases. The formation of the HCW subcommittee helped guide the design and conduct of the HERO Registry, a novel online registry of HCWs developed early in the COVID-19 pandemic. The subcommittee members’ diverse roles in healthcare and lived experiences helped to generate a balanced perspective for the HERO Registry Program and directly influenced aspects of its research efforts.

Prioritizing participant partner engagement with the research team enhances research recruitment and retention efforts as well as overall research quality. The HERO Registry’s HCW subcommittee provides an example of how lived experience partners can be integrated successfully into a research program and can provide valuable insights to shape and support participant-focused research.

## Supporting information

Chen-Lim et al. supplementary material Chen-Lim et al. supplementary material

## Supplementary material

The supplementary material for this article can be found at https://doi.org/10.1017/cts.2024.668.
