# Supplementary figures and images for: Through the lens of Good Participatory Practice: Findings and lessons learned from the healthcare worker subcommittee of the COVID-19 Healthcare Worker Exposure Response and Outcomes Registry
Source: J Clin Transl Sci. 2024 Dec 12;9(1):e9. doi: 10.1017/cts.2024.668 (PMC11736295; doi:10.1017/cts.2024.668)

Supplemental Figure: HERO Organizational Chart Figure

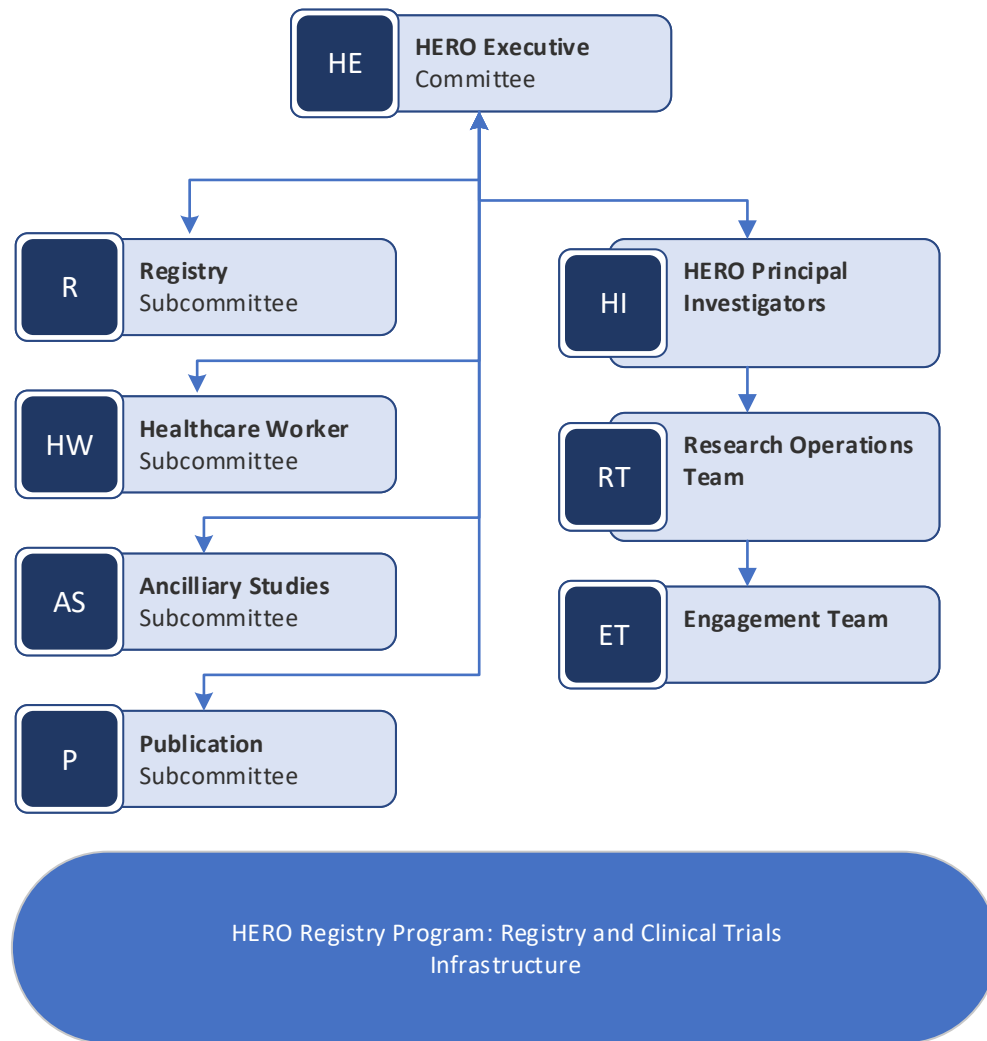

Supplement: Chen-Lim et al. supplementary material [file S205986612400668Xsup001.pdf]
